# Cord Blood IL-12 Confers Protection to Clinical Malaria in Early Childhood Life

**DOI:** 10.1038/s41598-018-29179-y

**Published:** 2018-07-18

**Authors:** Yong Song, Ruth Aguilar, Jing Guo, Maria Nelia Manaca, Augusto Nhabomba, Tamara Katherine Berthoud, Siew-Kim Khoo, Selma Wiertsema, Arnoldo Barbosa, Llorenç Quintó, Ingrid A Laing, Alfredo Mayor, Caterina Guinovart, Pedro L. Alonso, Peter N. LeSouëf, Carlota Dobaño, Guicheng (Brad) Zhang

**Affiliations:** 1 School of Public Health, Curtin University, Perth, 6102 Western Australia Australia; 2 Centre for Genetic Origins of Health and Disease, The University of Western Australia and Curtin University, Perth, 6009 Western Australia Australia; 3 Centro de Investigação em Saúde de Manhiça (CISM), Maputo, CP1929 Mozambique; 4 ISGlobal, Hospital Clínic of Barcelona, Universitat de Barcelona, Barcelona, Catalonia 08036 Spain; 5 School of Paediatrics and Child Health, The University of Western Australia, Perth, 6009 Western Australia Australia; 6 Curtin Health Innovation Research Institute, Curtin University, Perth, 6102 Western Australia Australia

## Abstract

Using a well-designed longitudinal cohort, we aimed to identify cytokines that were protective against malaria and to explore how they were influenced by genetic and immunological factors. 349 Mozambican pregnant women and their newborn babies were recruited and followed up for malaria outcomes until 24 months of age. Six Th1 cytokines in cord blood were screened for correlation with malaria incidence, of which IL-12 was selected for further analyses. We genotyped *IL-12* polymorphisms in children/mothers and evaluated the genotype-phenotype associations and genetic effects on IL-12 levels. Maternal IL-12 concentrations were also investigated in relation to *Plasmodium* infections and cord blood IL-12 levels. Our data showed that high background IL-12 levels were prospectively associated with a low incidence of clinical malaria, while IL-12 production after parasite stimulation had the opposite effect on malaria incidence. *IL-12* genotypes (*IL-12b* rs2288831/rs17860508) and the haplotype CGTTAGAG distribution were related to malaria susceptibility and background IL-12 levels. Maternal genotypes also exhibited an evident impact on host genotype-phenotype associations. Finally, a positive correlation in background IL-12 levels between maternal and cord blood was identified. Thus, cord blood background IL-12 concentrations are important for protecting children from clinical malaria, likely mediated by both genotypes (children&mothers) and maternal immunity.

## Introduction

Childhood malaria remains one of the leading causes of morbidity and mortality with 500, 000 deaths annually. According to the WHO World Malaria Report 2017^1^, more than 90% of malaria infections occurred in sub-Saharan Africa, of which 90% of deaths were attributable to *Plasmodium falciparum*. In malaria endemic regions, children under five years of age are highly susceptible to clinical malaria, while infants up to six months are relatively resistant, although the exact mechanism remains unclarified^2^. The host response to the parasite infection varies from asymptomatic carriage to the classic symptoms of clinical episode (e.g. fever, chills, sweating, headache and muscle aches), or to even severe life-threatening complications such as severe anaemia, respiratory distress, hyperparasitemia and cerebral malaria.

The basic immunity features of *Plasmodium* infections are pro-inflammatory responses in the early stage followed by anti-inflammatory responses during disease progression^3,4^. Pro-inflammatory Th1-type cytokines (IL-1, IL-6, IL-12, IFN-γ and TNF) are thought to be critical for controlling the erythrocytic and hepatic stages of *Plasmodium* infection, but the excessive production of these cytokines might also predispose to severe pathology^3–5^. Anti-inflammatory Th2-type and modulatory cytokines (IL-10 and TGF-β) downregulate the Th1-type cytokine response, thereby preventing subjects from severe forms of malaria^3,4,6^. Numerous immuno-epidemiological studies conducted in malaria endemic areas have illustrated the complex and paradoxical relationships between inflammatory cytokines and clinical presentations of *P. falciparum* infection^7–13^. For example, an elevated expression of IL-1, IL-6, IL-12, IFN-γ and TNF was found in severe malaria and in hyperparasitemia, a primary clinical feature of severe disease^7–10^. On the other hand, other studies showed that reduced IL-12 and IFN-γ levels were associated with an increased risk of severe malarial anaemia in children^11,12^, while lower levels of IL-6 were correlated with hyperparasitemia^9^.

Due to dynamic changes of cytokine profiles during physiopathological processes, most of the published studies using cross-sectional designs are not convincing to infer causal relationships of cytokine responses with disease phenotypes. A longitudinal birth cohort of infants is therefore important for understanding naturally acquired immunity to malaria and the associated genetic regulatory mechanisms^14–16^.

We hypothesised that Th1 cytokines at birth are important for determining the susceptibility of young children to clinical malaria. Using our well-defined prospective cohort^14–16^, we preliminarily screened six candidate Th1 cytokines (IL-1, IL-6, IL-12, IFN-γ, TNF and TNF-β), and identified IL-12 as a promising molecule for malaria protection. IL-12 is a heterodimeric p70 protein composed of p35 and p40 subunits encoded by *IL-12a* and *IL-12b* genes respectively. We further hypothesised that *IL-12* polymorphisms are associated with the disease protection during early life, through governing the functional expression of IL-12. Here we evaluated the associations of cord blood IL-12 concentrations, and *IL-12* genotypes/haplotypes with malaria outcome. Maternal effects on childhood malaria have been reported^17,18^, thus we also explored the significance of maternal regulation of cord blood IL-12 expression in modifying the disease susceptibility in the infant.

## Results

### Clinical and parasitological characteristics

General information on the children and their mothers included in this study is described in our previous publications^14–16^. Approximately 20% of the children were not successfully followed up or subjected to laboratory tests due to a variety of reasons such as parental withdrawal and insufficient blood collection. During the two-year follow-up, 108 children (108/326, 33.1%) had one or more malaria episodes. Twenty-three children (23/284, 8.1%) were diagnosed with anaemia at the endpoint of follow-up. Concurrently, 32 (32/284, 11.3%) and 101(101/256, 39.5%) children were identified as *P. falciparum* positive for parasitemia by microscopic method and qPCR assay, respectively. In terms of pregnancy infection, we found 27 (27/349, 7.7%) mothers presented with maternal parasitemia and 60 (60/277, 21.7%) mothers with placental infection, determined by microscopy and histology, respectively. Additionally 25 (25/254, 7.2%) newborns presented congenital infection, as detected by qPCR in filter papers, which was included as one of the confounding factors for adjustment in our analysis models.

### Cord cytokine and malaria phenotypes

Using Poisson regression analysis and taking into account the effects of the confounding factors e.g. intervention, mother’s age, use of insecticide-treated mosquito nets (ITNs), use of indoor residual spraying (IRS) and congenital infection (parasites in cord), we investigated the association between the concentration of six Th1 cytokines secreted by cord blood cells in culture supernatants and the incidence of clinical malaria in the second year of life in young children (Table 1). We found that the background and stimulated/specific IL-12 levels had an opposite association with disease onset (Table 1). A high background IL-12 level in cord blood was prospectively associated with a low incidence of clinical malaria (*p* = 0.020) (Table 1). In contrast, a high IL-12 production or a high specific IL-12 response after *P. falciparum* antigen stimulation was significantly related to an increased risk of clinical malaria (*p* < 0.001) (Table 1). The stimulated form of IFN-γ also demonstrated a positive association with high morbidity of childhood malaria (*p* = 0.010) (Table 1).

**Table 1:** Association of pro-inflammatory cytokines secreted by cord blood with the incidence of clinical malaria in the second year of life.

Cytokine		IRRs	95% CI		*p*
			Lower	Upper	
IL-1	Background	0.93	0.82	0.94	0.321
	Stimulated	0.96	0.83	1.11	0.564
	Specific	1.11	0.87	1.42	0.407
IL-6	Background	0.98	0.88	1.09	0.666
	Stimulated	0.97	0.86	1.08	0.517
	Specific	0.96	0.76	1.20	0.693
IL-12	Background	0.74	0.57	0.96	0.020
	Stimulated	1.61	1.24	2.09	<0.001
	Specific	1.46	1.23	1.73	<0.001
IFN-γ	Background	0.97	0.77	1.23	0.819
	Stimulated	1.48	1.10	1.99	0.010
	Specific	1.16	0.96	1.40	0.136
TNF	Background	0.91	0.78	1.06	0.207
	Stimulated	0.91	0.78	1.06	0.214
	Specific	1.01	0.80	1.27	0.957
TNF-β	Background	0.71	0.37	1.35	0.298
	Stimulated	1.04	0.71	1.52	0.847
	Specific	1.53	0.94	2.50	0.090

Poisson regression model was employed after adjusting for intervention, mother’s age, parity, infant sex, use of insecticide-treated mosquito nets, use of indoor residual spraying, and congenital infection. 95% CI: 95% confidence interval. Background: cytokine levels without stimulation (uninfected erythrocytes); Stimulated: cytokine production after *P. falciparum* schizont lysate stimulation; Specific: level of cytokine after stimulation divided by its background level. n = 240 for each group. IRR: Incidence rate ratio.

Further, we tested the association of cord cytokine levels with other malaria related phenotypes including parasitemia and anaemia. The specific IL-12 response was marginally higher in 2 year old children who had microscopically confirmed parasitemia (*p* = 0.054), relative to those without parasitemia (Supplementary Figure 1a). After controlling the aforementioned confounders, the difference showed statistical significance (*p* = 0.044) (Supplementary Table 1). As for anaemia, the background IL-1 levels were significantly lower in children with anaemia (*p* = 0.024) (Supplementary Table 1), whereas such difference was not evident for IL-12 (Supplementary Figure 1b). Using the Pearson correlation analysis, we found that the *Plasmodium*-specific IL-12 response was positively associated with blood *P. falciparum* copy number (r = 0.150, *p* = 0.036, n = 196) quantified by qPCR assay in filter paper samples. The specific IL-12 response was not correlated with plasma haemoglobin concentration. Moreover, a correlation between IL-1 levels and plasma haemoglobin concentration was not detected.

Apart from the associations outlined above, we did not observe any other significant differences (Supplementary Table 1). As IL-12 proved promising in preventing childhood malaria, our subsequent studies were focused on this cytokine, while the other cytokines were thereafter excluded from further analysis.

### Genotypes and haplotypes with malaria phenotypes

The distributions of the genotypes in young children and their mothers are summarized in Supplementary Table 2. Significant departure from the HWE was observed for children’s *IL-12b* rs2288831 (*p* = 0.025) and mothers’ *IL-12b* rs2546890 (*p* = 0.010) in the distribution of genotypes in the overall population. However, when we tested in the respective control groups (children without congenital infection and mothers without maternal parasitemia), such deviation was not significant for any of the examined single nucleotide polymorphisms (SNPs) in both children and mothers (*p* > 0.05).

**Figure 1:**
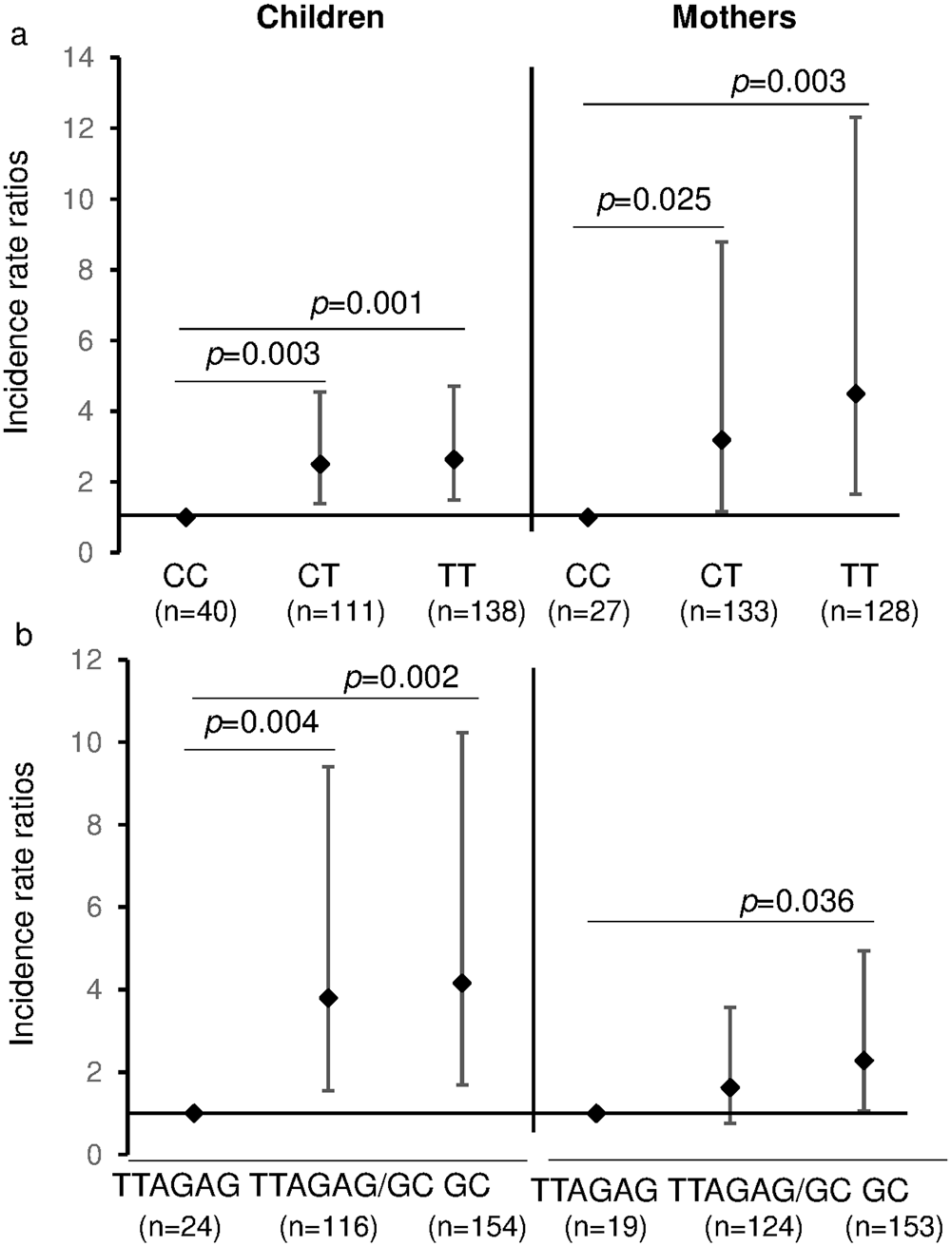
*IL-12b* Genotypes and Malaria Incidence: Incidence rate ratios were compared in host and maternal genotypes of *IL-12b* rs2288831 (**a**) and *IL-12b* rs17860508 (**b**). The homozygous genotypes CC and TTAGAG were used as reference allele for the two *IL-12b* polymorphisms respectively. Poisson regression analysis was used, controlling for intervention, mother’s age, parity, infant sex, use of insecticide-treated mosquito nets, use of indoor residual spraying, congenital infection. Values represent Mean (95% confidence interval).

Out of the four *IL-12* SNPs examined, the *IL-12b* rs2288831 and rs17860508 genotypes were found to be significantly associated with malaria incidence (*p* = 0.005 and *p* = 0.007 respectively) (Table 2). Children with allele C of *IL-12b* rs2288831 had a significantly lower incidence of malaria in the second year than those with allele T (CC vs CT, *p* = 0.003; CC vs TT, *p* = 0.001) (Fig. 1a). The GC allele of host *IL-12b* rs17860508 was associated with a high risk of clinical malaria occurrence compared to the TTAGAG allele (TTAGAG vs TTAGAG/GC, *p* = 0.004; TTAGAG vs GC, *p* = 0.002) (Fig. 1b). Similar effects were revealed for the maternal genotypes of *IL-12b* rs2288831 and rs17860508 and their association with clinical episodes of malaria (*p* < 0.001 and *p* = 0.005 respectively) (Table 2 and Fig. 1). Additionally a significant effect of the genotype distribution of maternal *IL-12b* rs2546890 on malaria incidence was identified (*p* = 0.008), with increasing risk from allele G to A (Table 2). After adjusting for the corresponding maternal SNPs, the effect of children’s *IL-12b* rs17860508 genotypes on malaria was decreased but remained significant (*p* = 0.023), while the significant effect of *IL-12b* rs2288831 disappeared (*p* = 0.929).

**Table 2:** Association of *IL-12* genotypes/haplotypes with the incidence of clinical malaria in the second year of life.

	n	IRRs	95%		*p*
			Lower	Upper	
Host genotypes					
*IL-12a* rs568408	294	0.91	0.72	1.15	0.428
(0: GG; 1: GA; 2: AA)					
*IL-12b* rs2288831	289	0.74	0.6	0.92	0.005
(0: TT; 1: TC; 2: CC)					
*IL-12b* rs17860508	294	0.73	0.58	0.92	0.007
(0: GC; 1: GC/TTAGAG; 2: TTAGAG)					
*IL-12b* rs2546890	292	0.75	0.79	1.19	0.782
(0: GG; 1: GA; 2: AA)					
Host haplotypes					
CGTTAGAG	281	0.58	0.36	0.95	0.031
TAGC	281	0.96	0.62	1.49	0.849
TGGC	281	1.86	1.25	2.78	0.002
Maternal genotypes					
*IL-12a* rs568408	299	0.78	0.6	1.01	0.059
(0: GG; 1: GA; 2: AA)					
*IL-12b* rs2288831	288	0.61	0.47	0.78	<0.001
(0: TT; 1: TC; 2: CC)					
*IL-12b* rs17860508	296	0.7	0.54	0.9	0.005
(0: GC; 1: GC/TTAGAG; 2: TTAGAG)					
*IL-12b* rs2546890	292	1.37	1.08	1.73	0.008
(0: GG; 1: GA; 2: AA)					

Poisson regression model was employed after adjusting for intervention, mother’s age, parity, infant sex, use of insecticide-treated mosquito nets, use of indoor residual spraying, congenital infection. 95% CI: 95% confidence interval. IRR: Incidence rate ratio.

**Figure 2:**
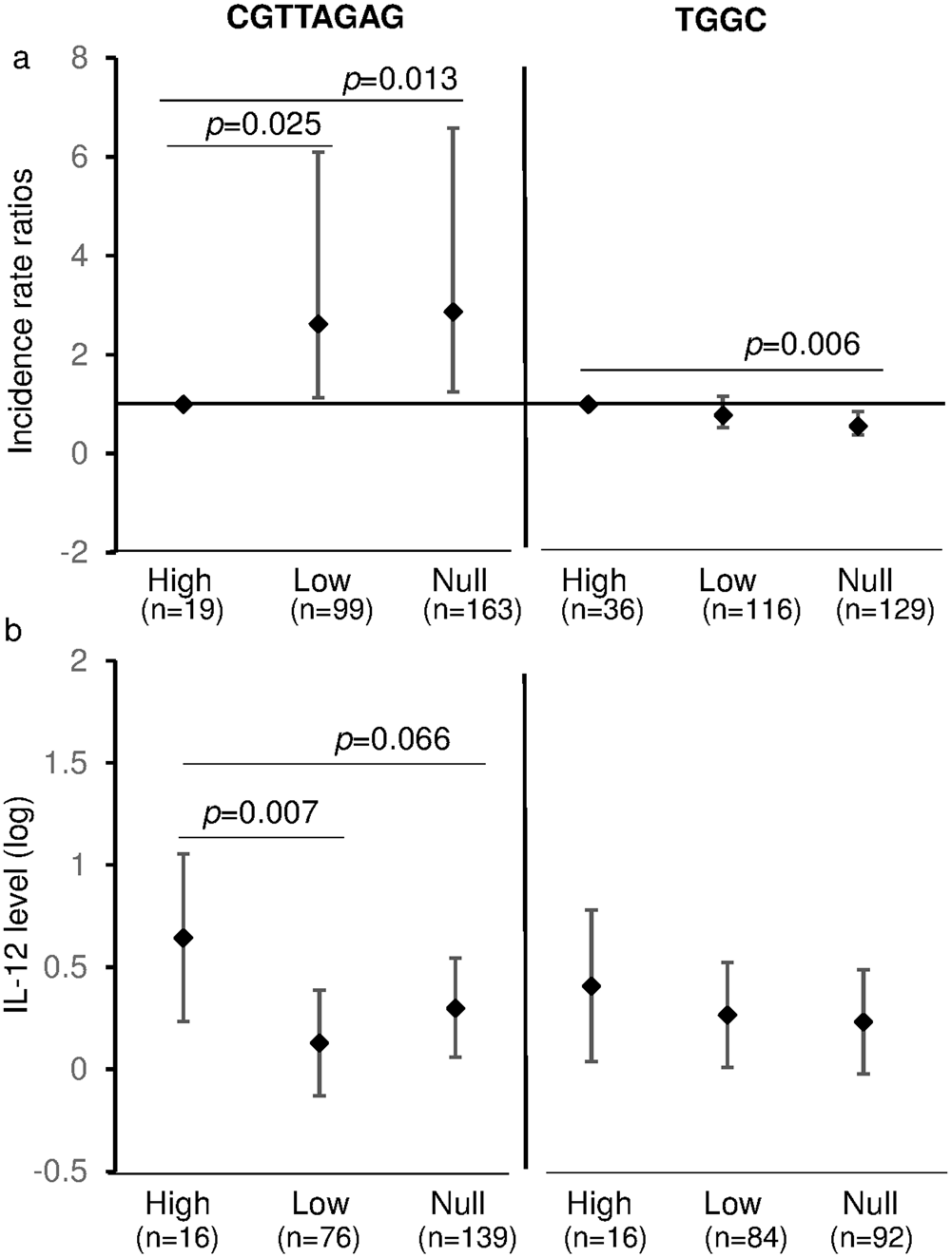
Children *IL-12b* Haplotypes and Malaria Incidence/Cord Blood IL-12 Production: Incidence rate ratios were compared in children with different probabilities of carrying the haplotypes CGTTAGAG and TGGC using Poisson regression model, with high probability as reference haplotype (**a**). The cord blood background IL-12 levels were stratified by different probabilities of carrying host haplotypes CGTTAGAG and TGGC using linear regression model (**b**). All analyses are adjusted for the confounding factors. High: 100% probability; Low: 20–50% probability; Null: zero probability. Values represent Mean (95% confidence interval).

A strong linkage was identified amongst *IL-12b* gene SNP rs2546890, rs2288831 and rs17860508 (Supplementary Figure 2), and the association of haplotypes with malaria episodes was subsequently investigated (Table 2). The host CGTTAGAG haplotype was significantly related to a decreased risk of malaria episodes during the first 2 years of life (High probability vs Low probability, *p* = 0.025; High probability vs Null, *p* = 0.013) (Fig. 2a), while newborns with a TGGC haplotype had an increased risk of malaria episodes (High probability vs Null, *p* = 0.006) (Fig. 2a). The haplotype TAGC did not alter the susceptibility to a clinical malaria episode.

Nevertheless, analysing genetic effects of *IL-12* on parasitemia and anaemia, we did not observe significant associations of either *IL-12* genotype or haplotypes with these malaria related phenotypes (Supplementary Table 3).

### Correlation of *IL-12* genotypes/haplotypes with IL-12 concentrations

**Figure 3:**
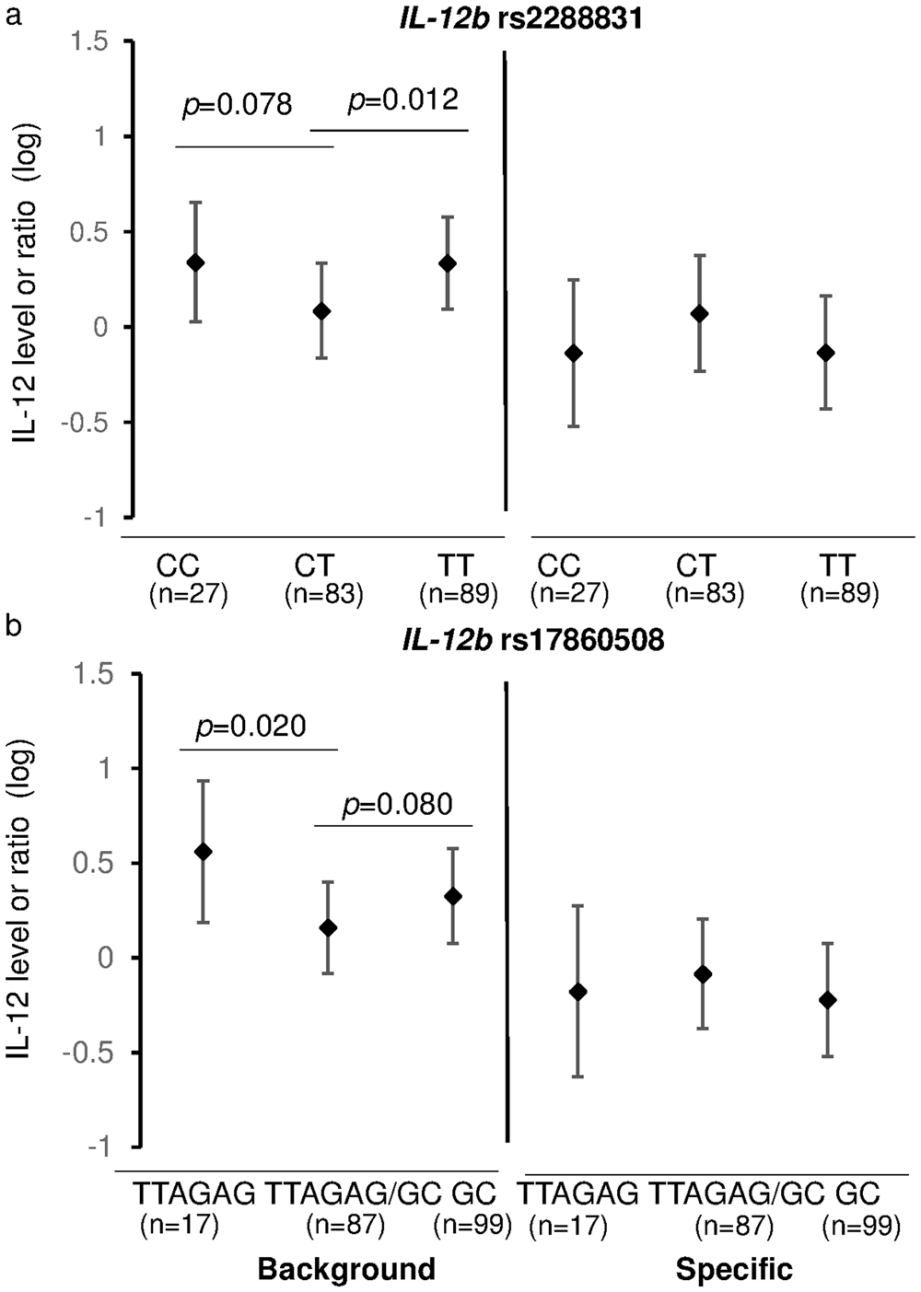
Cord Blood IL-12 Levels and Children *IL-12b* Polymorphisms: The cord blood background IL-12 levels and specific IL-12 production after *P. falciparum* schizont extract stimulation were stratified by children *IL-12b* genotypes of rs2288831 (**a**) and rs17860508 (**b**) using linear regression analysis with adjustment of the confounding effects. Values are Mean (SD).

IL-12 levels were significantly different in the genotypes of *IL-12b* rs2288831 (*p* = 0.030) and rs17860508 (*p* = 0.036) (Supplementary Table 4). Specifically, the background IL-12 concentrations were lower in children with heterozygous CT for *IL-12b* rs2288831 than those with homozygous CC (*p* = 0.078) and TT (*p* = 0.012) (Fig. 3a). Likewise, children with TTAGAG or GC genotype had significantly or marginally higher levels of background IL-12 than those with heterozygous TTAGAG/GC, respectively (Fig. 3b). Apart from this, we did not observe any other significant effects of host genotype on IL-12 levels.

The host CGTTAGAG haplotype had a significant effect on background IL-12 production (*p* = 0.018) (Supplementary Table 4). Low probability of CGTTAGAG haplotype was related to low background IL-12 levels and an increased risk of malaria episodes as outlined earlier (Fig. 2), consistent with the effects of the cord blood IL-12 levels on malaria outcome (Table 1).

### Impact of maternal immunity on cord blood IL-12 levels

**Figure 4:**
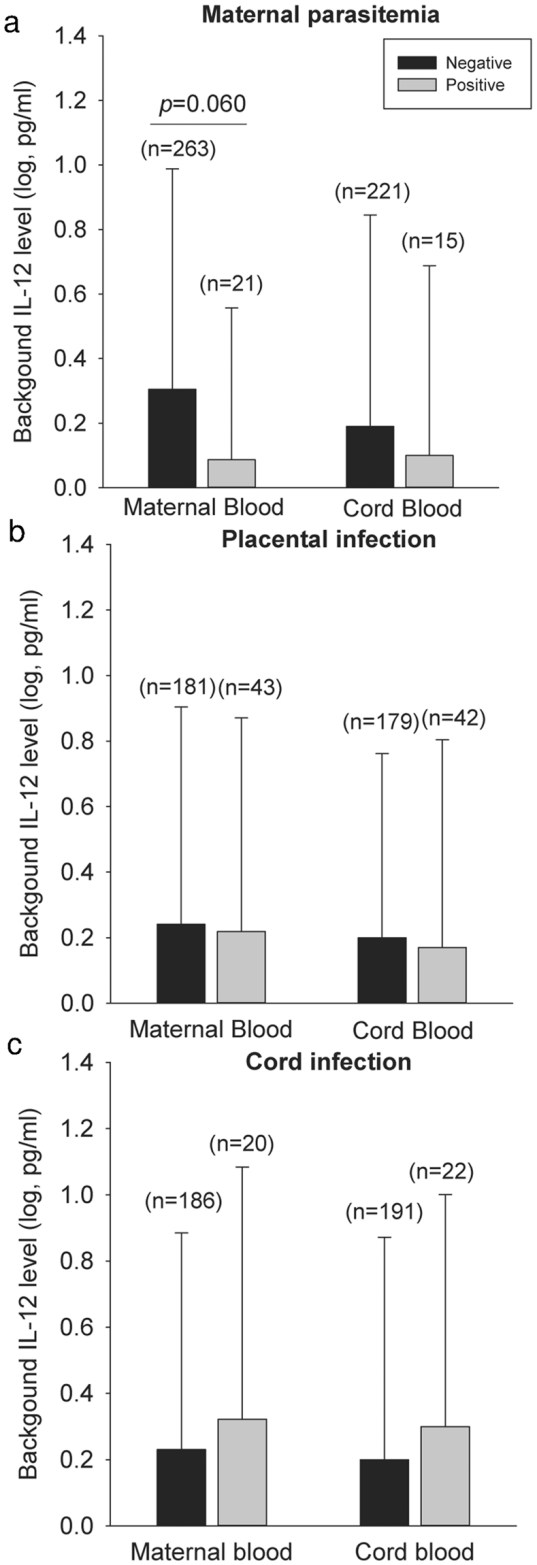
Maternal/Cord Blood IL-12 Production and Prenatal Malaria Infection: The background IL-12 levels in maternal peripheral blood and cord blood were compared in positive/negative maternal parasitemia (**a**), positive/negative placental infection (**b**) and positive/negative cord blood infection (**c**). Differences between the two groups are compared using independent *t* test. Values are Mean (SD).

The background levels of IL-12 in cord blood mononuclear cells were positively correlated with those in maternal peripheral blood mononuclear cells (PBMC) (r = 0.343, *p* < 0.001). However, there was no significant correlation between cord and maternal specific IL-12 production in response to antigen stimulation (r = 0.014, *p* = 0.845). Further analysis was performed to evaluate the impact of maternal malaria and congenital malaria on maternal and cord blood background IL-12 levels (Fig. 4). The background IL-12 concentrations in maternal peripheral blood were decreased in response to maternal parasitemia, although such change did not reach statistical significance (*p* = 0.060) (Fig. 4a). However, neither maternal nor cord blood background IL-12 levels differed in women with/without placental infection (Fig. 4b) or in newborns with/without congenital malaria (Fig. 4c)

## Discussion

Our study showed that children with a high background IL-12 expression in cord blood were highly resistant to the development of malaria episodes. We further established the relationships between host *IL-12* polymorphisms and incidence of childhood malaria, which was dependent on maternal genotypes. *IL-12* genotypes (*IL-12b* rs2288831 and rs17860508) and the haplotype CGTTAGAG distribution were related to the background IL-12 levels. In addition, we showed that the cord blood IL-12 levels were associated with the maternal IL-12 levels, which were affected by maternal malaria. Thus, we conclude that the background IL-12 levels in cord blood may be important for protecting young children from *Plasmodium* infection, likely mediated by the genotypes of children and mothers as well as maternal immunity.

Out of six Th1 pro-inflammatory cytokines investigated, we identified significant associations of IL-12 and IFN-γ at birth with the incidence of malaria infection in the first 2 years of life. Data from animal models of malaria indicated that an early response of both cytokines was essential for parasite clearance, but had to be maintained on a delicate balance to avoid inappropriate induction leading to malaria pathology^4^. Indeed, in the present study the relationship between these cytokines and disease phenotypes appears to be complex, particularly for IL-12. Specifically, we found that the background IL-12 levels were associated with protection of malaria onset, while *Plasmodium*-specific IL-12 production was associated with a high risk of malaria episodes, parasitemia and anaemia. However, given the double-edged effects of these cytokines, the contradictory associations are not so unexpected and are consistent with the previous reports^19,20^. Dodoo *et al*. demonstrated that the propensity of a person to secrete IL-12, IFN-γ, and TNF was associated with protection against parasitemia, clinical malaria, and anaemia^19^. On the contrary, the secretory IL-12 induced by the mitogen phytohemagglutinin or *Mycobacterium tuberculosis* antigen was negatively correlated to haemoglobin concentration^19^. Similarly, Riley *et al*. showed that IFN-γ production from PBMC in response to soluble exoantigens of *P. falciparum* was associated with increased risk of malaria symptoms^20^, supporting our findings that antigen-induced IFN-γ is a risk factor for increasing malaria susceptibility. Nevertheless, innate IFN-γ response to malaria antigens was reported to be stronger for live than for dead parasites and both γδ T cells and NK cells responded preferentially to live parasites^21^. These results raise the possibility that a protective IFN-γ response may derive from rapidly activated cell types rather than from memory T cells^19^. Thus, one of the limitations in the present study is that we investigated the cytokine production responding to schizont lysate, which may be different from live parasites^19^. However, an *in vivo* mouse study confirmed that infection with blood-stage lethal *P. berghei* NK65 induced IL-12 production, which was critically involved in the pathogenesis of liver injury via increasing IFN-γ^22^. Treatment of these infected mice with neutralizing monoclonal antibodies against IL-12 prolonged the survival and diminished liver injury^22^. Taken together, although further studies are required to ascertain the role of IL-12 production in response to the parasite infection, our data clearly demonstrated the importance of basal IL-12 levels in protecting children from *Plasmodium* infection. This finding has significant implications with respect to vaccine design strategy, e.g. up-regulating IL-12 expression in cells rather than directly supplying exogenous IL-12 to increase cellular resistance to infection.

The variation of cytokine production may be related to individual genetic differences^19^, affecting the outcome of *Plasmodium* infection. A functional promoter variant (rs17860508) in *IL-12b*, was found to have a dominant effect on cerebral malaria susceptibility in Tanzanian^23^ and Malian children^24^, and Thai population^25^, but not in Kenyan^23^ and Burkina Faso subjects^26^. In our cohort study, we found that heterozygous alleles of both *IL-12b* rs2288831 and rs17860508 were related to reduced expression of cord blood background IL-12 levels and a high risk of malaria episodes. These observations suggest that *IL-12b* polymorphisms, via regulation of baseline IL-12 production, influence the outcome of malaria infection. Nevertheless, our data showed that homozygous GC of *IL-12b* rs17860508 was associated with increased morbidity in 2-year-old Mozambican children, while others demonstrated that homozygosity for the CTCTAA allele affected susceptibility to cerebral malaria^24,25^. The apparent discrepancy may be explained by the different disease phenotypes observed (clinical episodes in the present study vs severe malaria in other studies^23–25^), particularly considering the different roles of cytokines in malaria progression, or due to the different ethnic groups studied.

Both homozygous alleles of TT and GC in *IL-12b* rs2288831 and rs17860508 were related to increased incidence of developing clinical malaria, compared to the homozygous alleles of CC and TTAGAG respectively. However, IL-12 levels were comparable between the two groups of homozygosity. We speculated that genetic variants were likely to form a regulatory haplotype to affect the function expression. As expected, construction of haplotypes for the three polymorphisms (*IL-12b* variants rs2546890, rs2288831 and rs17860508) revealed that carriage of the CGTTAGAG haplotype was associated with a reduced incidence of malaria episodes, while high probability of transmitting the TGGC haplotype was associated with increased susceptibility to the outcome of *Plasmodium* infection. Moreover, basal IL-12 concentrations were higher in children with the CGTTAGAG haplotype, confirming our hypothesis that the *IL-12b* haplotype exhibits a cis**-**regulatory effect on *IL-12* expression, contributing to disease protection. However, the distribution of the TGGC haplotype was not related to the cord blood IL-12 levels, and thereby the protective mechanism could not be explained by modulating IL-12 expression. Additional mechanisms (e.g. epistatic effects) may exist, as evidenced by genetic interactions of *IL-12* with *IL-10*^27,28^, haemoglobin C (*Hbc*)^29^ and toll-like receptor 4 (*TLR4*)^30^ in mediating IL-12 production or altering malaria related phenotypes.

Similar to the children’s genotypes, association of mothers’ genotypes with clinical malaria showed a similar pattern and consistent direction. Moreover, the impact of children’s genotypes on malaria episodes was attenuated markedly or disappeared, after adjusting for the corresponding maternal genotypes, suggesting a maternal genotype-dependent mechanism underlying host genotype-phenotype interactions. In addition, prenatal exposure to malaria is well documented to increase parasite density and severe malaria risk in offspring. Our data showed a positive correlation of background IL-12 levels between maternal and cord blood. Moreover, maternal malaria seemed to decrease the background IL-12 concentrations in the mother. As such, cord blood IL-12 levels may play a key role in transducing malaria signalling to modify infant susceptibility. It is speculated that owing to the passively acquired malarial antigens from maternal source, the fetal cell is primed and activated to produce the coordinative expression of Th1/Th2 phenotypes in fetal blood^31^.

In conclusion, we report for the first time that basal expression of IL-12 in cord blood cells confers protection from *Plasmodium* infection throughout early life. IL-12 genotype/haplotypes alter paediatric malaria susceptibility, which is in part associated with regulating IL-12 expression. Finally, we found that maternal genotype and IL-12 response were associated with the cord blood IL-12 levels and subsequent development of malaria disease in the offspring. It follows that interactions between maternal genetic factors and environment determine childhood malaria outcome, through altering the inherent expression of the cord blood IL-12.

## Materials and methods

### Participants

This study was part of the AgeMal project (ClinicalTrials.gov identifier NCT00231452) described previously^15^. The primary aim of this study was to investigate the effect of age of first exposure to malaria on the development of naturally acquired immunity in infants. In summary, the study was a three-arm randomized, double-blind, placebo-controlled trial carried out in a malaria endemic area (Manhiça district of Southern Mozambique) from August 2005 to July 2009. A total of 349 HIV-negative pregnant women and their newborn babies were recruited and followed up until two years of life after birth. The first exposure to *P. falciparum* was selectively controlled at varying postnatal stages (2 to 5 months, early exposure; 5 to 10 months, late exposure; or none, control) with monthly chemoprophylaxis with sulfadoxine-pyrimethamine and artesunate. However, such strategy did not affect outcomes of *P. falciparum* infection and antibody response^14,15^. Written informed consent was obtained from all mothers. The study was approved by the National Mozambican Ethics Review Committee, the Hospital Clinic of Barcelona Ethics Review Committee, and the Princess Margaret Hospital for Children Ethics Committee (1473/EP) in Perth. All experiments were performed in accordance with relevant guidelines and regulations of the Hospital Clinic of Barcelona and the School of Paediatrics and Child Health, the University of Western Australia.

### Cytokine measurement

Blood mononuclear cells were isolated from maternal and cord bloods using a lymphoprep gradient, and resuspended in complete RPMI medium. The fresh cells were stimulated with uninfected erythrocytes or *P. falciparum* schizont extract^32^. After 24 h culture, IL-1, IL-6, IL-12 (p70 heterodimer), IFN-γ, TNF and TNF-β levels were measured in the culture supernatants using the Bender MedSystems Human Th1/Th2 11plex FlowCytomix multiplex kit and analyzed on a FACS Canto II (Becton Dickinson, Franklin Lakes, NJ, USA)^32,33^. Cytokine concentrations (pg/ml) measured in this way represented background cytokines without stimulation (uninfected erythrocytes), or cytokine production in response to *P. falciparum*-specific stimulation. The secretory capacity of cytokines was further expressed by division of background cytokines from the corresponding stimulated cytokines (stimulated cytokine/background cytokine), defined as specific cytokine responses after malaria antigen stimulation.

### Disease phenotypic definitions

As described previously^15^, newborns were followed up weekly (from birth to 10.5 months) or monthly (from 10.5 to 24 months) for malaria case detection and in case of illness, subjected to clinical assessments and blood smear. The case detection included taking axillary temperatures and recording any history of fever. A clinical malaria episode was defined as axillary temperature ≥37.5 °C, or a history of fever within the prior 24 h plus the presence of *P. falciparum* of any density diagnosed by microscopy. Since the interventions were conducted in the first year of life, a clinical episode of malaria infection during the second year of life was the main phenotype in our analysis. Two other phenotypes, namely anaemia and parasitemia, were also assessed at 24 months of age. Anaemia was defined as haemoglobin <8 g/dl. For childhood parasitemia except assessed by microscopy, qPCR was also used to determine cord blood or peripheral blood *P. falciparum* copy number in filter paper samples at birth and 24 months of age. Concerning maternal malaria, maternal parasitemia was determined in peripheral blood by microscopy and placental infection was assessed by histology. The laboratory test details were described in our recent paper^32^.

### Genotyping

Genomic DNA from PBMC was purified using an automated DNA extraction instrument (Autopure LS; Qiagen, Hilden, Germany). We selected one SNP rs568408 in *IL-12a* gene 3′-UTR and three SNPS (*IL-12b* rs17860508, rs2546890, rs2288831) in *IL-12b* gene regions. Among these polymorphic markers, *IL-12a* rs568408, *IL-12b* rs17860508 and *IL-12b* rs2546890 were found to be related to malaria infections^24,25^, while *IL-12b* rs2288831 was shown to have a strong linkage disequilibrium with *IL-12b* rs17860508^26^. The four SNPs were genotyped by using the iPLEX assay on a MALDI-TOF MassARRAY platform (Sequenom, San Diego, CA, USA) in the Australian Genome Research Facility.

### Data analysis

The allele and genotype distribution was examined for the Hardy Weinberg Equilibrium (https://regepi.bwh.harvard.edu/IIPGA2/Bioinformatics/) and compared using chi square test. Linkage disequilibrium analysis was performed using Haploview v4.2 software.

Log-transformation was used to normalize the distribution of cytokine values. The associations between cytokine concentrations and disease phenotypes (e.g. anaemia, parasitemia or prenatal malaria) or genotypes/haplotypes were analysed using independent sample *t* test or one-way ANOVA, respectively. Linear regression was used to verify the associations in children after adjusting for the potential confounders e.g. mother’s age, parity, infant sex, ITNs, IRS, and congenital infection since these factors were known to influence cord cytokine profile and childhood malaria susceptibility^17,18,34,35^. The variable of intervention group (early, late exposure and control) was fitted in the regression model as an additional adjustment for analysing cytokine association with the malaria disease phenotypes. Poisson regression was used to determine the risk of the incidence of clinical malaria in the second year of life and its association with cytokine production and genotypes/haplotypes of *IL-12* with adjustment for the confounding factors outlined above. The Pearson correlation index was calculated to determine the association among different continuous variables using linear regression analysis.

SPSS (version 20.0: SPSS Inc., Chicago, IL, USA) was used for the statistical analyses. Data were presented as mean (95% CI or SD). Differences were accepted as significant at *p* < 0.05.

### Data availability

The datasets generated during and/or analysed during the current study are available from the corresponding authors on reasonable request.

## Electronic supplementary material

Supplementary Information
