# Supplementary material for: Cord Blood IL-12 Confers Protection to Clinical Malaria in Early Childhood Life
Source: Sci Rep. 2018 Jul 18;8:10860. doi: 10.1038/s41598-018-29179-y (PMC6052074; doi:10.1038/s41598-018-29179-y)
Supplement: Supplementary file 1 — Supplementary Information [file 41598_2018_29179_MOESM1_ESM.pdf]

Cord Blood IL-12 Confers Protection to Clinical Malaria in Early Childhood Life

Running head: IL-12 and Clinical Malaria

Yong Song[1,2], Ruth Aguilar[3,4], Jing Guo[1,2], Maria Nelia Manaca[3], Augusto Nhabomba[3], Tamara Katherine Berthoud[4], Siew-Kim Khoo[5], Selma Wiertsema[5], Arnoldo Barbosa[3], Llorenç Quintó[4], Ingrid A Laing[5], Alfredo Mayor[4], Caterina Guinovart[3,4], Pedro L. Alonso[3,4], Peter N. LeSouëf[5], Carlota Dobaño[3,4], Guicheng (Brad) Zhang[1,2,6*]*

[1] *School of Public Health, Curtin University, Perth, 6102, Australia*

[2] *Centre for Genetic Origins of Health and Disease, The University of Western Australia and Curtin University, Perth, 6009, Australia*

[3] *Centro de Investigação em Saúde de Manhiça (CISM), Maputo, CP1929, Mozambique*

[4] *ISGlobal, Hospital Clínic of Barcelona, Universitat de Barcelona, Barcelona, Catalonia, 08036, Spain*

[5] *School of Paediatrics and Child Health, The University of Western Australia, Perth, 6009, Australia*

[6] *Curtin Health Innovation Research Institute, Curtin University, Perth, 6102, Australia*

[*] Equal senior authors; Address correspondence to:

Carlota Dobaño; Address: ISGlobal, Carrer Rosselló 153 (CEK building), E-08036 Barcelona, Catalonia, Spain; Tel: +34 93 2275400 ext. 4519; Email: Carlota.dobano@isglobal.org.

Guicheng (Brad) Zhang; Address: School of Public Health, Curtin University of Technology, Kent St, Bentley, Western Australia 6102, Australia; Tel: +61 8 9266 3226; Fax: +61 8 9266 9266; Email: brad.zhang@curtin.edu.au.

Supplementary Table 1. Associations of Th1 proinflammatory cytokines with malaria phenotypes

		Parasitemia			Anaemia		
		**Negative**	**Positive**	***p***	**Negative**	**Positive**	***p***
**IL-1**	Background	2.23 (1.94-2.64)	2.14 (1.59-2.69)	0.562	2.32 (1.98-2.67)	1.75 (1.18-2.31)	**0.024**
	Stimulated	2.26 (1.93-2.59)	2.31 (1.80-2.82)	0.827	2.31 (1.98-2.63)	1.87 (1.34-2.41)	0.070
	Specific	-0.03 (-0.23-0.17)	0.17 (-0.15-0.48)	0.169	-0.02 (-0.22-0.18)	0.13 (-0.20-0.45)	0.328
**IL-6**	Background	2.79 (2.34-3.24)	2.89 (2.18-3.59)	0.763	2.83 (2.38-3.28)	2.53 (1.80-3.26)	0.360
	Stimulated	2.842 (2.425-3.259)	3.004 (2.352-3.657)	0.583	2.88 (2.47-3.29)	2.70 (2.02-3.38)	0.548
	Specific	0.05 (-0.16-0.26)	0.12 (-0.21-0.45)	0.659	0.05 (-0.16-0.26)	0.17 (-0.18-0.51)	0.442
**IL-12**	Background	0.28 (0.05-0.52)	0.18 (-0.18-0.55)	0.544	0.28 (0.05-0.52)	0.13 (-0.25-0.51)	0.368
	Stimulated	0.22 (-0.04-0.49)	0.51 (0.09-0.92)	0.132	0.25 (-0.02-0.51)	0.38 (-0.05-0.82)	0.482
	Specific	-0.06 (-0.33-0.21)	0.32 (-0.10-0.74)	**0.044**	-0.04 (-0.31-0.23)	0.25 (-0.19-0.62)	0.142
**IFN-γ**	Background	0.21 (0.01-0.43)	0.16 (-0.18-0.50)	0.729	0.20 (-0.02-0.41)	0.25 (-0.10-0.61)	0.734
	Stimulated	0.19 (-0.07-0.44)	0.21 (-0.19-0.60)	0.913	0.18 (-0.07-0.43)	0.27 (-0.15-0.68)	0.651
	Specific	-0.02 (-0.29-0.24)	0.05 (-0.36-0.46	0.695	-0.02 (-0.28-0.24)	0.01 (-0.42-0.44)	0.878
**TNF**	Background	1.21 (0.88-1.53)	0.92 (0.41-1.42)	0.204	1.20 (0.88-1.52)	0.86 (0.33-1.39)	0.149
	Stimulated	1.31 (0.99-1.63)	1.13 (0.62-1.63)	0.432	1.30 (0.98-1.62)	1.16 (0.63-1.69)	0.550
	Specific	0.10 (-0.12-0.33)	0.21 (-0.14-0.56)	0.482	0.10 (-0.13-0.32)	0.30 (-0.07-0.66)	0.222
**TNF-β**	Background	0.15 (0.01-0.28)	0.06 (-0.16-0.27)	0.337	0.15 (0.01-0.28)	0.04 (-0.18-0.26)	0.284
	Stimulated	0.12 (-0.03-0.26)	0.13 (-0.09-0.35)	0.894	0.12 (-0.02-0.26)	0.13 (-0.10-0.36)	0.911
	Specific	-0.03 (-0.14-0.07)	0.07 (-0.09-0.24)	0.150	-0.03 (-0.13-0.07)	0.09 (-0.08-0.26)	0.119

Linear regression model was used after adjusting for intervention, mother's age, parity, infant sex, use of insecticide-treated mosquito nets, use of indoor residual spraying, congenital infection. Values are Mean (95% confidence interval).

Supplementary Table 2. Genotype frequencies in infants and mothers

	Infants			Mothers		
	**No. (%)**	**MAF**	**HWE (*p*)**	**No. (%)**	**MAF**	**HWE (*p*)**
**Genotypes**						
***IL-12a* rs568408**						
**GG**	172 (58.5)	A: 23.3%	0.754	176 (58.9)	A: 23.2%	0.960
**GA**	107 (36.4)			107 (35.8)		
**AA**	15 (5.1)			16 (5.4)		
***IL-12b* rs2288831**						
**TT**	138 (47.8)	C: 33.0%	**0.025**	128 (44.4)	C: 32.5%	0.367
**TC**	111 (38.4)			133 (46.2)		
**CC**	40 (13.8)			27 (9.4)		
**rs17860508**						
**GC**	154 (52.4)	TTAGAG: 27.9%	0.743	153 (51.7)	TTAGAG: 27.4%	0.354
**GC/TTAGAG**	116 (33.2)			124 (41.9)		
**TTAGAG**	24 (8.2)			19 (6.4)		
**rs2546890**						
**GG**	129 (44.2)	A: 34.15	0.585	134 (45.9)	A: 30.3%	**0.010**
**GA**	127 (43.5)			141 (48.3)		
**AA**	36 (12.3)			17 (5.8)		

MAF, minor allele frequency; HWE, Hardy-Weinberg equilibrium.

Supplementary Table 3. Associations of genotypes / haplotypes with disease phenotypes

		Parasitemia			Anaemia		
**Genotypes**	**N**	n (%)	**OR**	***p***	n (%)	**OR**	***p***
IL-12a rs568408							
GG	147	15 (10.2%)	1.00	0.138	9 (6.1%)	1.00	0.859
GA	96	14 (14.6%)	2.57		9 (9.4%)	1.25	
AA	14	0 (0.0%)	0.00		2 (14.3%)	1.70	
IL-12b rs2288831							
TT	116	14 (12.1%)	1.00	0.636	11 (9.5%)	1.00	0.744
CT	100	11 (11.0%)	1.33		8 (8.0%)	1.36	
CC	37	3 (8.1%)	0.63		2 (5.4%)	0.75	
rs17860508							
GC	134	18 (13.4%)	1.00	0.605	13 (9.7%)	1.00	0.959
TTAGAG/GC	101	9 (8.9%)	0.68		7 (6.9%)	0.92	
TTAGAG	21	2 (9.5%)	0.43		1 (4.8%)	0.73	
rs2546890							
GG	115	13 (11.3%)	1.00	0.973	5 (4.3%)	1.00	0.290
AG	112	12 (10.7%)	0.94		14 (12.5%)	2.44	
AA	28	3 (10.7%)	0.83		1 (3.6%)	0.84	
**Haplotypes**							
CGTTAGAG							
Null	138	18 (13.0%)	1.00	0.657	12 (8.7%)	1.00	0.884
Low	91	7 (7.7%)	0.63		7 (7.7%)	1.33	
High	16	2 (12.5%)	0.62		1 (6.3%)	1.18	
TAGC							
Null	118	13 (11.0%)	1.00	0.968	6 (5.1%)	1.00	0.451
Low	106	11 (10.4%)	0.95		13 (12.3%)	2.01	
High	21	3 (14.3%)	1.19		1 (4.8%)	0.94	
TGGC							
Null	90	9 (10.0%)	1.00	0.931	6 (6.7%)	1.00	0.639
Low	98	8 (8.2%)	1.02		6 (6.1%)	1.69	
High	32	3 (9.4%)	1.30		6 (18.8%)	0.00	

OR: odds ratio. The associations (overall *p* value) were investigated using logistic regression analyses between SNPs and disease phenotypes, adjusted by intervention, mother's age, parity, infant gender, use of insecticide-treated mosquito nets, use of indoor residual spraying, congenital infection. Null: zero probability; Low: 20-50% probability; High: 100% probability.

Supplementary Table 4. Associations of cord blood IL-12 levels with host genotypes / haplotypes

	Background	*p*	Stimulated	*p*	Specific	*p*
IL-12a rs568408						
GG	0.19 (-0.06, 0.44)	0.658	0.12 (-0.16, 0.40)	0.532	-0.07 (-0.36, 0.22)	0.400
GA	0.20 (-0.06, 0.46)		0.24 (-0.04, 0.53)		0.04 (-0.26, 0.32)	
AA	0.377 (-0.032, 0.786)		0.125 (-0.328, 0.577)		-0.252 (-0.728, 0.224)	
IL-12b rs2546890						
GG	0.23 (-0.01, 0.48)	0.847	0.20 (-0.08, 0.47)	0.927	-0.04 (-0.33, 0.26)	0.957
GA	0.19 (-0.07, 0.44)		0.15 (-0.14, 0.40)		-0.03 (-0.30, 0.21)	
AA	0.26 (-0.08, 0.59)		0.17 (-0.21, 0.55)		-0.09 (0.49, 0.31)	
rs2288831						
TT	0.33 (0.09, 0.58)	**0.030**	0.20 (-0.08, 0.48)	0.916	-0.14 (0.43, 0.16)	0.205
TC	0.08 (-0.16, 0.33)		0.15 (-0.13, 0.44)		0.07 (-0.24, 0.37)	
CC	0.338 (0.025, 0.651)		0.201 (-0.159, 0.561)		-0.137 (-0.521, 0.248)	
rs17860508						
GC	0.325 (0.076, 0.574)	**0.036**	0.102 (-0.178, 0.383)	0.274	-0.22 (-0.522, 0.08)	0.489
GC/TTAGAG	0.158 (-0.082, 0.399)		0.07 (-0.20, 0.34)		-0.09 (-0.38, 0.202)	
TTAGAG	0.559 (0.185, 0.933)		0.38 (-0.04, 0.80)		-0.18 (-0.63, 0.27)	
CGTTAGAG						
Null	0.300 (0.056, 0.543)	**0.018**	0.12 (-0.16, 0.40)	0.398	-0.18 (-0.48, 0.11)	0.472
Low	0.128 (-0.130, 0.386)		0.07 (-0.23, 0.36)		-0.06 (-0.37, 0.25)	
High	0.643 (0.234, 1.052)		0.37 (-0.10, 0.83)		-0.28 (-0.77, 0.22)	
TAGC						
Null	0.258 (0.004, 0.513)	0.949	0.13 (-0.16, 0.41)	0.886	-0.13 (-0.43, 0.17)	0.978
Low	0.235 (-0.026, 0.496)		0.08 (-0.21, 0.37)		-0.15 (-0.46, 0.16)	
High	0.286 (-0.101, 0.673)		0.15 (-0.28, 0.59)		-0.13 (-0.59, 0.33)	
TGGC						
Null	0.232 (-0.023, 0.487)	0.529	0.17 (-0.11, 0.46)	0.335	-0.06 (-0.36, 0.24)	0.406
Low	0.265 (0.009, 0.520)		0.04 (-0.24, 0.33)		-0.22 (-0.52, 0.08)	
High	0.409 (0.038, 0.780)		0.27 (-0.14, 0.69)		-0.14 (-0.58, 0.30)	

Linear regression model was used after adjusting for mother's age, parity, infant gender, use of insecticide-treated mosquito nets, use of indoor residual spraying, congenital infection. Values are Mean (95% confidence interval). Null: zero probability; Low: 20-50% probability; High: 100% probability.

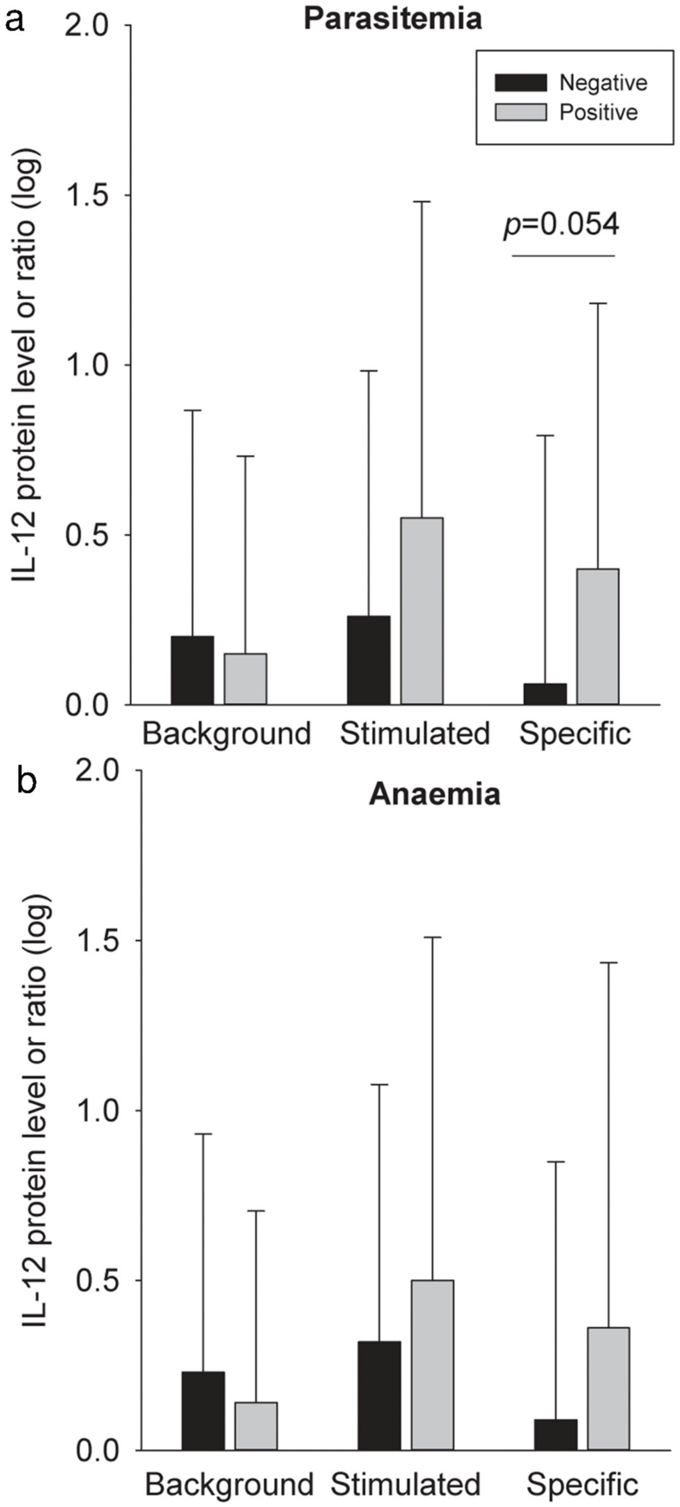

Figure 1 – Cord Blood IL-12 Production and Malaria Phenotypes: The cord blood cell background IL-12 concentrations, stimulated IL-12 and specific IL-12 supernatant production in response to *P. falciparum* schizont extract were compared in positive (n=176) / negative (n=20) parasitemia (a) or positive (n=177) / negative (n=19) anaemia (b) in children at age 2 years old. Differences between the two groups were compared using independent *t* test. Values are Mean (SD).

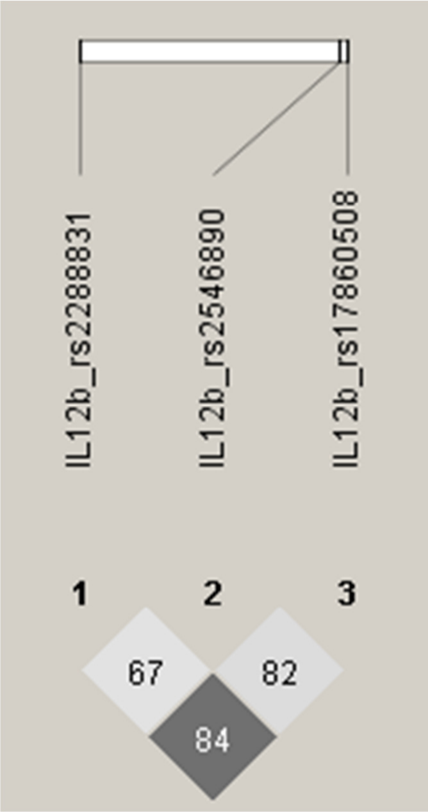

Supplementary Figure 2: – Linkage Disequilibrium of *IL-12* Genetic Polymorphisms in Children: The Linkage disequilibrium (LD) for the three single nucleotide polymorphisms (*IL-12b* rs2546890, rs2288831 and rs17860508) in children was examined and constructed using Haploview version 4.2. High pairwise LD between polymorphic markers is illustrated with dark shading. The r^2 values (×100) for the marker pairs are listed in the corresponding boxes.
